# An Analysis of the Hypothalamic-Pituitary-Adrenal Axis Functions in Cirrhotic Rats in Response to Surgical Stress

**DOI:** 10.1155/2018/7606304

**Published:** 2018-06-28

**Authors:** Fahimeh Yarigholi, Ali Zare Mehrjardi, Zahra Azizi, Massoud Baghai Wadji

**Affiliations:** ^1^Department of Surgery, Musavi Hospital, Zanjan University of Medical Science, Zanjan, Iran; ^2^Department of Pathology, Firoozgar Hospital, Iran University of Medical Science, Tehran, Iran; ^3^Department of Surgery, Firoozgar Hospital, Iran University of Medical Science, Tehran, Iran

## Abstract

**Introduction:**

The activation of hypothalamic-pituitary-adrenal (HPA) axis through severe diseases and stress courses leads to a rise in circulatory cortisol for an adequate response to stress. This axis is one of the important systems that involve in neuroendocrine response to the surgical stress. Hepatoadrenal syndrome that is a manifestation of adrenal insufficiency (AI) in the course of liver disease is described as insufficient production of steroid hormones mainly cortisol due to primary dysfunction of the adrenal gland or secondary malfunction of the HPA axis to provoke the adrenal gland leading to severe illness and increased mortality. Through this evidence, we presented this question as to whether cirrhotic patients have a greater mortality rate than other patients after surgery and if the HPA axis is partly responsible for this phenomenon. Also how the adrenal gland functions during surgery in cirrhotic rats. We conducted this study to assess the effect of cirrhosis on the HPA axis through surgery in cirrhotic rats by evaluating the changes in serum corticosterone level and blood sugar before, immediately, and 30 minutes after surgery.

**Method:**

This study was performed in the animal lab approved by the Ethics Committee of Tehran University of Medical Sciences in 2014, on 25 male Wistar rats. Thioacetamide was used for induction of cirrhosis in rats with new method of monitoring weekly changes of rats' weight which had 100% success in procedure and reduction in mortality rate. Laparotomy was performed on all of the rats during 9–12 in the morning within 10–15 minutes. Laparotomy was chosen as surgical stress because of its simplicity and feasibility. Three blood samples were obtained from each rat immediately after inducing anesthesia, immediately after the conclusion of surgery, and 30 minutes after surgery. The plasma concentration of corticosterone was measured with enzyme-linked immunosorbent assay test. *P* value of 0.05 or less was considered as statistical significance.

**Result:**

Cirrhotic rat group consisted of 15 rats and control group consisted of 10 rats. There was a significant difference in the mean level of corticosterone and blood sugar between the cirrhotic rat group and control group in the 3 time levels (*P*=0.044/*P* < 0.001). Pairwise comparison of mean corticosterone and blood sugar levels between case (mean: 249.359 ± 3.90) and control (mean: 262.40 ± 4.69) showed a significant difference (*P*=0.04, 95% CI = 0.30–25.79/*P* < 0.001, 95% CI = 129.62–233.96). Unlike the control group, the level of serum corticosterone was compared in the cirrhotic rat group (group 1) before, immediately, and 30 minutes after surgery, which showed a significant difference in our study (*P* value  = 0.005). However, this result was also significant in comparing the blood sugar in 3 time levels of surgery in the control group (*P* value < 0.001) but not in the cirrhotic rat group (*P* value = 0.233).

**Conclusion:**

There was a significant rise in corticosterone levels during 3 time levels of surgery in cirrhotic rats; nevertheless, this elevation was significantly lower than the control group. Also the mean level of blood sugar was higher in the control group than in cirrhotic rats. However, this difference was significant in comparison with the same times of surgery between the two groups. These results approximately can substantiate our hypothesis that AI in the field of cirrhosis would also affect the response of HPA axis to stress during and after surgery that can be concomitant with higher rate of cardiovascular unsteadiness incidences, deteriorating the severity of illness and rise in mortality rate.

## 1. Introduction

Hypothalamic-pituitary-adrenal (HPA) axis functions as a set of physiologic reactions involving hypothalamus, pituitary, and adrenal gland to adjust physiological functions such as response to stress, modulating immune system reactions, and digestion [1–5]. Among many studies conducted regarding functions of the HPA axis, cortisol has been known as the most potent and influential immunosuppressive hormone which has a vital function in stressful events such as modulation of the inflammatory responses and cytokine production in severe sepsis [1, 3, 5–13]. The activation of HPA axis through severe diseases and stress courses leads to a rise in circulatory cortisol for an adequate response to stress [5, 9, 14, 15]. Adrenal insufficiency (AI) that is characterized by inadequate production of steroid hormones, mainly cortisol, is a disorder that can be seen in acute illnesses such as trauma, severe infection, and sepsis and infrequently after surgery [5, 8, 9, 11–13, 16, 17]. Insufficient cortisol response to stress and relative adrenal insufficiency are associated with poor prognosis and hemodynamic inconsistency such as vasopressor-dependent refractory hypotension with high cardiac output and reduced systemic vascular resistance after surgery leading to an increased mortality rate [7, 9, 13, 17–20]. The occurrence of AI in the course of liver disease or cirrhosis is called “hepatoadrenal syndrome” and is associated with a higher incidence of cardiovascular unsteadiness, severe illness, increased mortality rate, and liver transplantation [8, 10, 12, 13, 15, 21–23].

A number of studies have been conducted in this context to evaluate adrenal function in patients with liver failure or cirrhosis to utilize adrenal function parameters as prognostic factors in the course of the disease. The data from these studies suggest a positive correlation between the degrees of adrenal dysfunction with severity of liver disease [13, 21]. Furthermore, other studies have reported the increased level of corticosterone throughout surgery, anesthesia, and postoperative course, but yet no studies have been able to investigate the changes in the function of the HPA axis before and after surgery in cirrhotic patients [1, 6–8, 13, 14, 24, 25]. As a result, this study was designed to evaluate the function of HPA axis in response to surgical stress in cirrhotic rats compared with noncirrhotics by measuring corticosterone and blood sugar levels before and after surgery.

## 2. Method

### 2.1. Animals

This study was performed on 25 male Wistar rats aged between 8 and 10 weeks with a body weight of 200–250 grams. Five plexiglass cages (RAZI RAD Industries, Tehran, Iran) with standard condition were used to house the rats. Each cage contained 5 rats. Cages were cleaned once a week.

Temperature and humidity of the room were about 22 ± 2°C and 30–70%, respectively, and air conditioning was about 10 times per hour. The light and dark cycle was adjusted to 12-hour light and 12-hour dark.

All the experiments on animals were approved by the Ethics Committee of Tehran University of Medical Sciences (TUMS).

### 2.2. Method of Inducing Cirrhosis

Various methods can be utilized to induce cirrhosis, namely, ligation of common bile duct or using hepatotoxins such as carbon tetrachloride (CCl4) and thioacetamide (TAA) along with weekly monitoring of weight changes [26]. In this study, we used thioacetamide for inducing cirrhosis in rats and monitored weekly changes of their weight. This method had 100% success and reduced mortality rate.

For every 100 gram weight of a rat's weight, 10 grams of food (Dehparvar Food Industry, Iran) was considered. The amount of food was equal in both groups, and all had free access to food. The rats in the control group received tap water. A daily distribution of water was available for the control group while the case group received drinking water containing 0.03% thioacetamide (Tarvand Sina, Isfahan, Iran) and had free access to water, 3 times a week with the aim of inducing cirrhosis. The rats in the case group were weighed weekly, and thioacetamide levels were adjusted according to their weight. If their weight exceeded 275 grams, they were moved to a separate cage and received water containing 0.045% thioacetamide. If the rats' weight became less than 200 grams, they were transferred to a separate cage and received a thioacetamide solution of 0.015%. After gaining weight ranging between 200 and 275 grams, they were moved back to their first cage. Thioacetamide solutions with the concentration of 0.015, 0.03, and 0.045 percent were prepared by solving 150, 300, and 450 milligrams of solid thioacetamide in 1000 cc water, respectively.

The rats were inspected thrice a week in terms of general health. Both groups were provided with the same living conditions, food, temperature, and illumination throughout the study period. In the 13th week of the study, one of the rats in the case group died. Within a period of 16 weeks, rats were successfully cirrhotic.

### 2.3. Anesthesia and Laparotomy

Laparotomy was performed on all rats between 9 and 12 in the morning. Each of the rats was weighed before surgery, and the amount of analgesic drugs was calculated according to their weight. In order to induce anesthesia, a mixture of lidocaine 1% (Lignodic 1% AMP, Caspian Tamin Pharmaceutical Company, Iran) and ketamine hydrochloride (ROTEXMEDICA, ketamine injection 50 mg/ml, 10 ml, Germany) was injected with a ratio of 0.1 cc and 0.25 cc, respectively, into their peritoneal cavity. After controlling the depth of anesthesia, the first sample of blood was obtained from the ophthalmic vein from the corner of the eye. Then, rats were laid in a supine position, their abdomen was shaved, and preoperative prepping was carried out with Betadine—povidone-iodine 10% (BETADINE® Solution, toluidine 10% 1 L solution, Tolid Daru Company, Iran) before surgery. A lower midline incision of 2.5 cm in length was made below the xiphoid process through the skin and abdominal muscles. The viscera and peritoneum were then manipulated by the surgeon with sterile gauzes for about 1 minute, and 2 cc normal saline was injected into the peritoneal cavity. Then, both the muscle layer and skin were sutured. The surgical procedure for each rat was completed within 10–15 minutes.

### 2.4. Sampling

Three blood samples (about 1 cc) were obtained from each rat. The first blood sample was obtained immediately after inducing anesthesia, and before incision, from the ophthalmic vein, using a capillary tube. The second was obtained immediately after the conclusion of surgery, from the vena cava. The third sample was obtained 30 minutes after the second sample from the heart area. Though the blood sampling was performed in different ways due to its feasibility, it was same in both control and case groups. All samples were obtained when the rats were still under complete anesthesia. The second and third samples were obtained using a special 2 cc syringe for animal experiments. Finally, the blood samples were centrifuged at 2000–3000 rpm and stored at −25 Celsius. The plasma concentration of corticosterone was measured using ELISA (enzyme-linked immunosorbent assay) test by ELISA kit from Glory Science Company, Hong Kong, China.

After all samples were obtained and while the rats were still anesthetized, the case group rats were reopened to obtain a liver biopsy specimen of 1 cm × 1 cm, for the pathologic conformation of cirrhosis. And also one biopsy specimen was obtained randomly from the control group. At the end of the procedure, all the rats were euthanized using chloroform gas.

### 2.5. Statistical Analysis

Statistical analyses were done by using the SPSS 22.0 software. Descriptive data were reported as mean ± SD (range). Statistical tests were two-tailed, and all the results with *P* value of 0.05 or less were considered as statistical significance. The comparison between 2 dependent quantitative results was accomplished using paired-sample *t*-test, and for data that were not normally distributed, the Wilcoxon test was performed. To compare the result of the level of corticosterone and blood sugar in different times, repeated-measures test (ANOVA, generalized linear model) was performed, and if there was a difference, it was followed by paired-sample *t*-test. Repeated measures with one within-subject and one between-subjects effect were conducted to compare the level of corticosterone and blood sugar before, immediately, and 30 minutes after surgery between case and control groups, and the Bonferroni test was used as a post hoc test for multiple pairwise comparisons between groups.

Independent *t*-test was done for comparing the level of blood sugar and corticosterone between the same times of surgery in each group (S0 in case and control, S1 in case and control, and S2 in case and control and C0 in case and control, C1 in case and control, and C2 in case and control).

We conducted one-way repeated-measures ANOVA to compare the level of serum corticosterone and blood sugar in the cirrhotic rat group (group 1) and control group separately during the 3 time levels of surgery (before, immediately, and 30 minutes after surgery). LSD (least significant difference) test was conducted as a post hoc test.

## 3. Results

**Figure 1:**
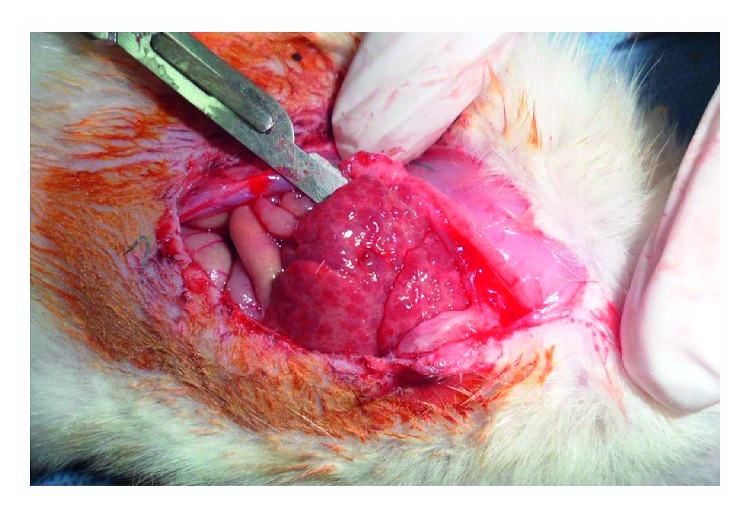
Picture of one of the cirrhotic rats showing diffuse nodular liver.

This study was done on 25 male Wistar rats with an average age between 8 and 10 weeks that were randomly divided into two groups. Cirrhotic rat group consisted of 15 rats and control group consisted of 10 rats. In the pathological findings of the liver biopsy specimen in the cirrhotic rat group, macronodular changes were reported, confirming successful induction of cirrhosis of all rats in the case group.  Figure 1 shows the gross nodularity of the liver in a cirrhotic rat. In analytical statistics, repeated measures with one within-subject and one between-subjects effect were conducted to compare the level of corticosterone and blood sugar before, immediately, and 30 minutes after surgery between case and control groups, and the Bonferroni test was used as a post hoc test for multiple pairwise comparisons between groups.

**Figure 2:**
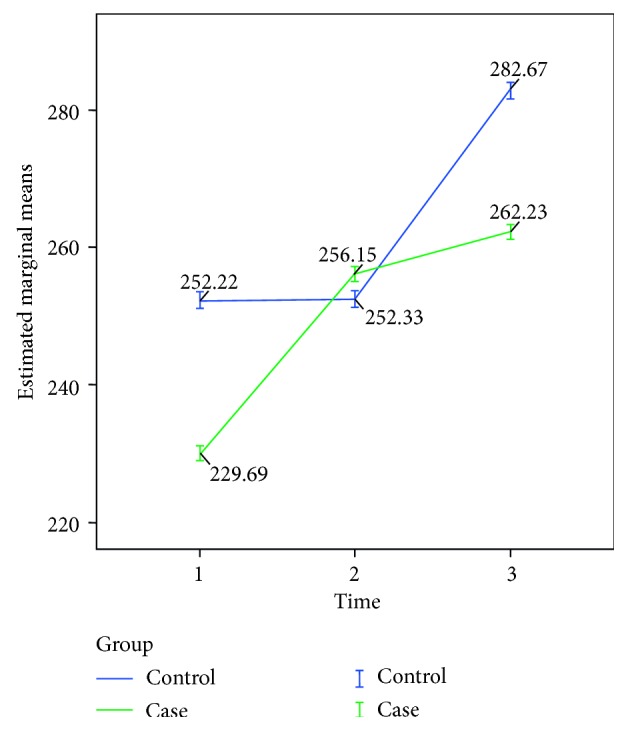
Mean level of serum corticosterone (microgram per deciliter) before, immediately, and 30 minutes after surgery between the cirrhotic rat group and control group.

There was a significant difference in the mean level of corticosterone between the cirrhotic rat group and control group in the 3 time levels in the test within subjects (*P*=0.044, *F* = 3.376, df = 2). The Bonferroni test for pairwise comparison of mean corticosterone levels between case (mean: 249.359, SD: 3.907, 95% CI: 241.210–257.508) and control (mean: 262.407, SD: 4.695, 95% CI: 252.613–272.202) groups showed a significant difference between corticosterone levels (*P*=0.045, *F* = 4.564, df = 1, 95% CI = 0.307–25.790) (Figure 2).

**Figure 3:**
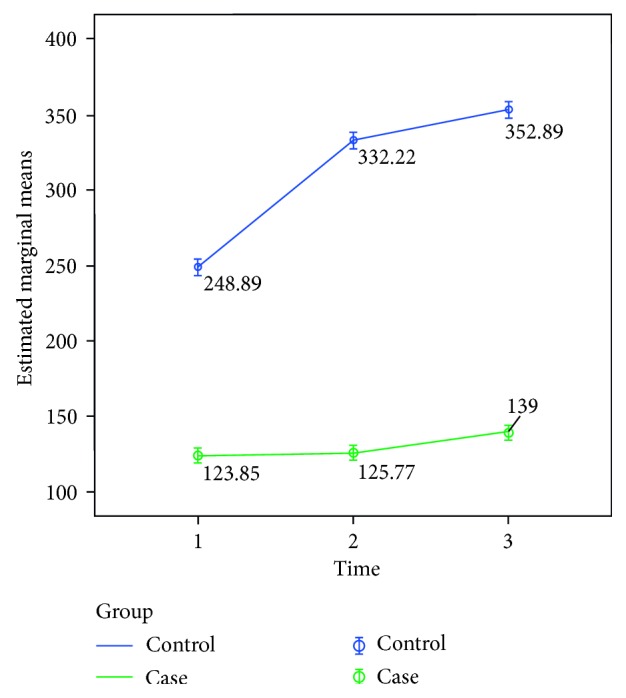
Mean level of serum sugar (milligram per deciliter) before, immediately, and 30 minutes after surgery between the cirrhotic rat group and control group.

Also, the mean level of serum blood sugar between the two groups in 3 times of measurement in the test within subjects was shown to have a significant difference in our study (*P* < 0.001, *F* = 19.378, df = 1.544). Bonferroni tests for pairwise comparison of mean blood sugar levels between the case group rats (mean: 129.538, SD: 15.995, 95% CI: 96.173–162.904) and control group rats (mean: 311.333, SD: 19.224, 95% CI: 271.233–351.434) showed a significant difference between serum blood sugar levels (*P* < 0.001, SE = 25.008, *F* = 52.845, df = 1, 95% CI = 129.629–233.961) (Figure 3).

In comparing the level of blood sugar between the same times of surgery in each group by independent *t-*test (S0 in case and control, S1 in case and control, and S2 in case and control), the control rat group had a higher level of blood sugar in comparison with the cirrhotic rat group in all three times (S0: *P*=0.002 and 95% CI = 59.531–187.818; S1: *P* < 0.001 and 95% CI = 162.396–260.266; S2: *P* < 0.001 and 95% CI = 136.710–285.862). But the corticosterone levels were not significantly different between cirrhotic and control groups.

We conducted one-way repeated-measures ANOVA to compare the level of serum corticosterone in the cirrhotic rat group (group 1) before, immediately, and 30 minutes after surgery, which showed a significant difference in our study (*P* value = 0.005, *F* = 6.790, df = 2). LSD (least significant difference) test was conducted as a post hoc test. There was a significant increase in the level of serum corticosterone immediately (*P* value = 0.002, SE = 6.509, 95% CI = 12.280–40.643) and also 30 minutes (*P* value = 0.007, SE = 10.102, 95% CI = 10.529–54.548) after surgery compared to its level before surgery in the cirrhotic rat group. However, when compared between two time levels of surgery (immediately and after 30 minutes), the amount of corticosterone did not show a significant change (*P* value = 0.589, SE = 10.958, 95% CI = −29.953–17.799).

The level of serum corticosterone in the control group showed an increase among the 3 time levels of surgery, but none of these differences were statistically significant (*P*=0.426, *F* = 0.900, df = 2).

One-way repeated-measures ANOVA for comparing the level of serum sugar in the cirrhotic rat group (group 1) before, immediately, and 30 minutes after surgery did not show a significant difference in our study (*P* value = 0.233, *F* = 1.584, df = 1.166).

By one-way repeated-measures ANOVA, the changes in serum blood sugar in the control group were significantly different before, immediately, and 30 minutes after surgery (*P* value < 0.001, *F* = 32.948, df = 2) with significant increase immediately (*P* value < 0.001, SE = 11.198, 95% CI = 57.511–109.155) and 30 minutes after surgery (*P* value < 0.001, SE = 15.050, 95% CI = 69.295–138.705) compared to the level before surgery. However, the level of serum sugar did not show a significant change between two time levels of surgery (immediately and after 30 minutes after surgery) (*P* value = 0.182, SE = 14.146, 95% CI = −53.288–11.954).

## 4. Discussion

The activation of HPA axis in severe illnesses, such as trauma, anesthesia, major surgery, burns, and sepsis, is the main mechanism for adjustment of homeostasis in the body and interaction between immune and adrenal system, neuronal stimulation of CRH, release of adrenocorticotropic hormone (ACTH), cortisol, and a variety of cytokines which are essential for a sufficient response to stress [1, 2, 5, 9, 12, 14, 20, 27]. Adrenal insufficiency was first described as a relative or absolute deficiency of glucocorticoids and mineralocorticoids that can be either a primary dysfunction of the adrenal gland or secondary malfunction of the HPA axis to provoke the adrenal gland and manifest as hypotension, hyponatremia, hyperkalemia, hypercalcemia, hypoglycemia, metabolic acidosis, eosinophilia, reduced response to catecholamine therapy, impairment in circulatory and renal function, hemodynamic unsteadiness, and higher mortality rate [5, 9, 10, 13, 23]. Adrenal insufficiency is a common condition in patients with advanced cirrhosis and septic shock [12, 13, 21]. This condition in liver disease has been described as hepatoadrenal syndrome [10, 21–23, 28]. Cortisol has an essential role in the synthesis of catecholamines and vasoactive peptides, which leads to a preservation of vascular tone and permeability to endogenous and exogenous stimulus and also the enhancement of vascular and cardiac reaction in severe sepsis and other conditions [5, 9, 10, 12, 13, 15]. Plasma corticosterone levels will be increased due to a stress response by induction of anesthesia and surgery [1]. Major surgeries such as cardiac surgery result in the activation of HPA axis due to development of systemic inflammation [27]. The level of stress response is associated with the severity of damage [1]. Through this evidence, we presented this question as to whether cirrhotic patients have a greater mortality rate than other patients after surgery and if the HPA axis is partly responsible for this phenomenon. Also how the adrenal gland functions during surgery in cirrhotic rats. We conducted this study to assess the effect of cirrhosis on the HPA axis through surgery in cirrhotic rats by evaluating the changes in serum corticosterone level and blood sugar before, immediately, and 30 minutes after surgery. Comparison of the results of mean level of serum corticosterone and blood sugar between the two groups in the study revealed a significant difference between the cirrhotic rat group and control group. There was a significant rise in corticosterone levels during 3 time levels of surgery in cirrhotic rats; nevertheless, this elevation was significantly lower than the control group.

Also the mean level of blood sugar was higher in the control group than in the cirrhotic rat group. However, this difference was significant in comparison with the same times of surgery between the two groups.

These results approximately substantiate our hypothesis that AI in the field of cirrhosis would also affect the response of HPA axis to stress during and after surgery. Also it can be concomitant with a higher rate of cardiovascular incidences, deteriorating health, increased severity of illness, and rise in mortality rate [8, 10, 12, 13, 15, 21–23]. Since the liver is the main site for metabolism of cortisol, a decrease in liver function or inhibition of glucocorticoid metabolism may result in a compromised clearance of cortisol in liver disease and cirrhosis [20, 22, 29, 30]. Goldkuhl et al. [1] measured the level of corticosterone during different time levels of surgery in male rats while they received lidocaine and buprenorphine for analgesia. The result of their study showed an association of surgical severity and type of analgesic treatment on the level of corticosterone through surgery, but none of them have reached the objectives of this study. Lausten et al. [6] demonstrated the result of open and laparoscopic cholecystectomy on hepatic stress response in patients with postnecrotic liver cirrhosis and chronic hepatitis. Besides other results, the changes of cortisol in both groups between pre- and postoperative time were not significant in this study. Banani and Omrani [24] compared the effect of surgical stress on HPA axis function in thalassemic and nonthalassemic patients. Their study showed a significant increase in cortisol response after surgery than the baseline in both groups.

Some hypotheses have been proposed to explain the mechanism of hepatoadrenal syndrome. Studies have revealed the role of bile acids in adjusting glucocorticoid metabolism in the liver and the impact of increasing bile acid levels in HPA axis resulting in adrenal insufficiency due to liver disease [20]. Cortisol can be decreased due to adrenal hemorrhage occurring in septic shock and also liver failure. Adrenal hypoperfusion due to circulatory impairment in cirrhosis may also have a role. Some studies have discussed the low levels of HDL cholesterol as a risk factor for adrenal dysfunction in liver disease. Since cortisol is not stored in the adrenal gland, a decrease in the level of HDL cholesterol may cause a lack of substrate for synthesis of cortisol during major stress. Furthermore, an increase in the level of bacterial endotoxin and other products in cirrhosis leads to the elevation of proinflammatory cytokines that have an impact on the function of the adrenal glands [8, 10, 13, 19, 22, 30]. High INR and postprandial plasma glucose, low serum protein, total cholesterol, and HDL cholesterol have been discussed as predictors of AI occurrence in liver failure [30].

However, the data in this study show impairment of HPA axis function in the cirrhotic group that can be a probable explanation of more postsurgical mortality in cirrhotic patients, but we wish to interpret the results cautiously due to limitation of this study including the small numbers of the rats, difficulty in diagnosis of AD in cirrhosis due to the delayed onset and offset of cortisol rhythm in cirrhosis, the different shape of cortisol peaks in each individual, not measuring other elements taking part in this phenomenon including catecholamines, appropriate interleukins, or ACTH, and other multifactorial reasons that make it a controversial issue [8, 19].

## 5. Conclusion

There was a significant rise in corticosterone levels during 3 times of blood sampling after anesthesia and after surgery in cirrhotic rats; nevertheless, this elevation was significantly lower than the control group. Also the mean level of blood sugar was higher in the control group than in cirrhotic rats. However, this difference was significant in comparison with the same times of surgery between the two groups. These results approximately can substantiate our hypothesis that AI in the field of cirrhosis would also affect the response of HPA axis to stress during and after surgery that can be concomitant with higher rate of cardiovascular unsteadiness incidences, deteriorating the severity of illness and rise in mortality rate.

From another point of view, HPA axis malfunction itself in cirrhotic groups which undergo a surgery can be a probable cause of higher mortalities.

Further studies following this preliminary study can illuminate more details of functions of these sophisticated systems in cirrhotic patients.
